# Effects of vibroacoustic stimulation on hypertension: a pilot cross-over experimental study

**DOI:** 10.1007/s40520-026-03447-8

**Published:** 2026-07-07

**Authors:** Sophie Saller, Francesca Fornasini, Italo Balzani, Filippo Ceccato, Federica Limongi, Leonardo Franz, Stefania Maggi, Leonardo Molinari, Marianna Noale, Nicola Bernardini, Caterina Trevisan, Alois Saller

**Affiliations:** 1 Department of Medical Sciences, University of Ferrara, Ferrara, Italy; 2 General Medicine, Sant’Antonio Hospital, Padua, Italy; 3 Emergency Medicine, Sant’Antonio Hospital, Padua, Italy; 4 Endocrinology Unit, University of Padua, Padua, Italy; 5 National Research Council (CNR), Aging Branch – Institute of Neuroscience, Padua, Italy; 6 Department of Neurosciences, Otolaryngology Section, University of Padua, Padua, Italy; 7 Santa Cecilia Conservatory of Music, Rome, Italy; 8 Associazione Italiana di Medicina Termale (AMIITTF), Bologna, Italy

**Keywords:** Hypertension, Vibroacoustic stimulation, Music therapy, Cross-over studies, Pilot projects

## Abstract

**Background:**

Arterial hypertension is a frequent condition, affecting up to one-quarter of the global adult population. Both pharmacological and non-pharmacological treatments are recommended for hypertension management. Among the latter, recent studies have shown the positive effects of music therapy; however, evidence on the impact of vibroacoustic stimulation (VAS) is still limited.

**Objective:**

This pilot study aimed to explore the feasibility and effects of a VAS intervention on blood pressure (BP) and other cardiopulmonary and stress-related parameters in patients with hypertension.

**Methods:**

A pilot single-sequence cross-over experimental study was conducted on 11 male adults with mild to moderate hypertension. Patients underwent two sessions: on Day 1, they lay for 30 min without any vibratory or musical stimuli. On Day 2, they were placed on the vibroacoustic bed delivering 40 Hz vibration for 30 min, followed by 7 min of vibration combined with relaxing music. Physiological and stress-related parameters were measured at multiple time points during both sessions.

**Results:**

Preliminary analysis showed no statistically significant differences between sessions. However, on Day 2, a more pronounced trend toward reductions in systolic and diastolic BP, heart and respiratory rate, and perceived stress was observed. A higher heart rate variability was recorded on Day 1 compared to Day 2.

**Conclusion:**

The VAS intervention appears feasible and may influence cardiovascular health parameters. Further studies with larger sample sizes are necessary to confirm these effects.

## Introduction

Arterial hypertension is a prevalent and growing public health concern. Globally, it affects approximately one out of three adults aged 30–79 years (≈ 1.4 billion people), with prevalence increasing markedly with age [1]. Hypertension is associated with an elevated risk of cardiovascular morbidity and mortality, particularly in older adults, making effective blood pressure (BP) control a major priority in preventive medicine [2, 3]. From a mechanistic perspective, hypertension results from complex interactions between genetic susceptibility and environmental factors, leading to sustained increases in systemic vascular resistance and/or cardiac output through sympathetic overactivity, renin–angiotensin–aldosterone system activation, renal sodium retention, endothelial dysfunction, vascular remodeling, and arterial stiffness [4]. In particular, modifiable risk factors of hypertension include excess body weight, high dietary sodium intake, physical inactivity, unhealthy diet, harmful alcohol use, smoking, psychosocial stress, and comorbidities such as diabetes and chronic kidney disease [1, 4].

Recently, lifestyle modifications have received growing attention as a form of primary prevention for cardiovascular risk, including hypertension [5–7]. In this context, mind–body breathing-based approaches have been associated with improvements in parameters related to BP and quality of life of hypertensive patients [8], as well as music therapy, which has emerged as a complementary intervention with benefits in stress reduction [9–12] and BP control [11].

A recent study showed that musical auditory stimulation can enhance the autonomic response to antihypertensive medications in well-controlled hypertensive patients [13]. Vlachopoulos et al. [14] found that classical and rock music acutely reduce arterial stiffness in healthy individuals, affecting vascular compliance and pulse wave harmonization. In a related study, Vanderley et al. [15] in young healthy women found that heavy metal reduced parasympathetic heart rate control, while classical music had no significant effects. A meta-analysis of 42 randomized controlled trials on perioperative music interventions reported reductions of pain in 13 studies and of heart rate and BP in 27% of the included trials [16]. However, the results across studies remain inconsistent, which may be attributed to heterogeneity in music delivery methods (live vs. passive vs. active listening), genres, and protocols used for outcome assessment. Moreover, it remains unclear whether music’s cardiovascular benefits are mediated by direct autonomic modulation, or by stress reduction, and whether there is a ‘dose-response’ relationship as a function of music parameters such as duration or volume.

A more structured approach to integrating sound and vibration is vibroacoustic stimulation (VAS), which combines low-frequency sinusoidal vibration with music. VAS has shown potential benefits across somatic and functional conditions, including pain reduction [16], relief of fibromyalgia symptoms [17, 18], and modulation of cardiovascular and autonomic parameters such as blood pressure (BP) and heart rate [19]. In the context of hypertension, VAS may be an adjunct rather than a substitute for antihypertensive drugs, potentially targeting stress-related sympathetic activation and adherence-related barriers that often limit BP control in daily practice. In particular, in the present study, we selected a low-frequency protocol centered on 40 Hz, as this frequency has been repeatedly used in vibroacoustic applications and is considered feasible and well tolerated while providing a clearly perceivable, rhythmically stable stimulus [20]. The session duration was chosen to match commonly adopted clinical protocols (approximately 10–45 min across studies) and to allow detection of acute physiological responses without imposing excessive burden on participants.

The rationale for expecting BP-related changes after VAS is grounded in plausible neurophysiological mechanisms. By simultaneously engaging auditory and somatosensory pathways, VAS may influence central networks involved in stress and autonomic regulation (including limbic–hypothalamic circuits) and promote rhythmic entrainment of cardiorespiratory dynamics. In turn, this may reduce sympathetic arousal and/or facilitate parasympathetic engagement, leading to transient reductions in vascular tone and BP. In parallel, a relaxation response could attenuate hypothalamic–pituitary–adrenal (HPA) axis activity, consistent with reductions in salivary stress hormones. Importantly, changes in HRV may reflect entrainment-related modulation of autonomic oscillations rather than a simple shift toward increased vagal tone.

Accordingly, this pilot study was designed to assess the feasibility and explore the acute effects of a single VAS session on BP and a set of cardiovascular, respiratory, and stress-related markers in male adults with mild-to-moderate hypertension. Given the limited evidence base and heterogeneity of prior protocols, an exploratory within-subject design was chosen to generate effect-size estimates and inform the design of adequately powered controlled trials.

Despite some heterogeneity in instruments and protocols for VAS administration [21], no serious adverse effects related to this intervention have been reported. Mild side effects like drowsiness, dizziness, or nausea may occur after the first session but usually resolve quickly [22]. Wigram and Grocke have noted precautions and contraindications, including acute inflammation, pacemakers, psychosis, pregnancy, hypotension, and individual hypersensitivity to vibration [23]. Despite these, VAS remains a non-invasive, relaxing, and well-tolerated therapy [24].

This pilot study aimed to assess the feasibility and the acute change in systolic and diastolic BP during VAS versus control (primary outcome) in adults with mild-to-moderate hypertension.

Secondary outcomes included changes in cardiorespiratory parameters, perceived stress, and salivary cortisol/cortisone. Given the limited evidence and heterogeneity of prior protocols, an exploratory within-subject design was chosen to generate effect-size estimates useful to plan adequately powered controlled trials.

## Methods

### Study design

This was a single-center, observational feasibility study with a single-sequence cross-over design. The study was conducted in accordance with the Declaration of Helsinki and approved by the Clinical Research Ethics Committee (CESC) at its meeting of September 14, 2023 (code 154537). All participants gave their informed written consent to participate in the study.

### Participants

Eleven male volunteers aged 45–65 years, diagnosed with stage 1 hypertension, were included. Exclusion criteria included psychiatric or neurological disorders, chronic pain, contraindications to VAS, pacemaker, atrial fibrillation, substance abuse, or the use of autonomic-acting medications. Beta-blockers were discontinued when present and replaced with ACE inhibitors or ARBs. Participants were consecutively enrolled from the General Medicine outpatient clinic of the Sant’Antonio Hospital, a tertiary referral center for cardiovascular autonomic function testing in autonomic neuropathy and head-up tilt testing for syncope assessment.

### Intervention and control

**Fig. 1:**
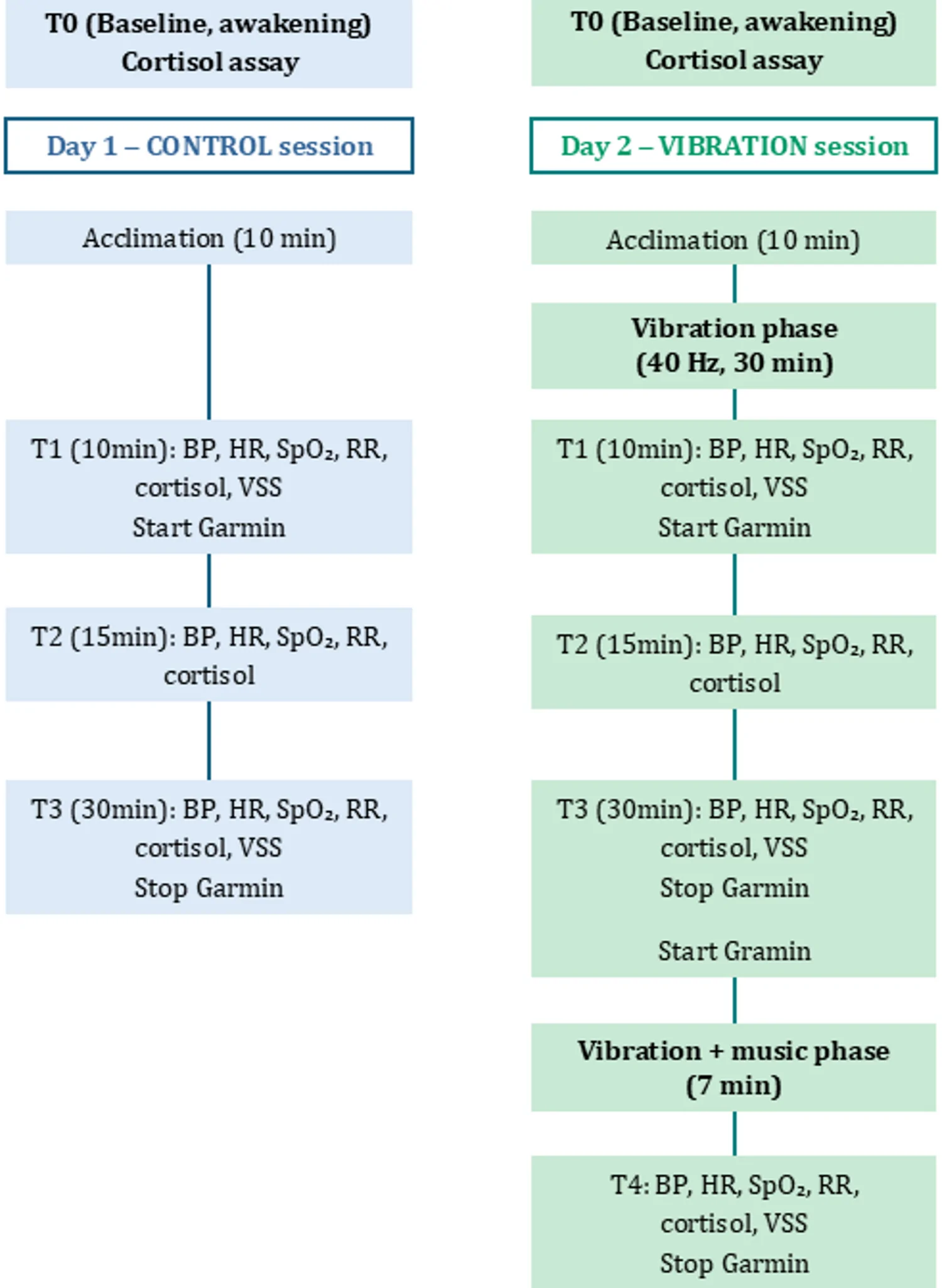
**Study protocol and measurement timeline.** Abbreviations: BP blood pressure, SpO₂ oxygen saturation, HR heart rate, RR respiratory rate, VSS visual stress intensity scale. T0 upon awakening; T1 after 10 min acclimatization; T2 mid-session 15 min after beginig control or treatment session; T3 end of control or treatment session; T4 after 7 min of music plus vibration

The structure of this pilot cross-over study is illustrated in Fig. 1.

On Day 1 (control session), participants, after 10 min of acclimation lying supine on a standard bed, lay in silence for 30 min.

On Day 2 (intervention session), after 10 min of acclimation, participants underwent VAS for 30 min at 40 Hz, followed by 7 min of combined vibration and music (60–80 bpm at ~ 50 dB). The intervention was delivered using the Vibbro 4 Massage 2.0 device, a vibroacoustic treatment bed developed by Vibbro Srl (https://vibbro.com/wp/). It consists of a harmonic wooden platform activated by three exciters (two covering 30–15,000 Hz and one for 20–120 Hz). The table vibrates freely and transfers vibrations to the contact surface through acoustic structures (soundpost and bridge). The frequencies are delivered via an application, the Vibbro Software Platform (VPS), developed by Vibbro Srl with musicians to reproduce tones within the audible range (20–15,000 Hz). The vibrations are partly transformed into acoustic sound by the resonating wood surface. To prevent temporary threshold shifts (TTS) or auditory fatigue from prolonged static sound [25], the system generates complex amplitude modulations using pure tone combinations. These modulations produce rhythmic amplitude variations, reducing fatigue.

### Data collection

Demographic, clinical (including the list of medications chronically used), laboratory, and echocardiographic data were collected at the enrollment visit. Age and smoking status (current smokers, n and %) were obtained from medical records and patient interviews. Body mass index (BMI, kg/m²) was calculated as weight divided by height squared. Blood samples were collected after overnight fasting and analyzed in the hospital central laboratory to determine hemoglobin (g/L), platelet count (×10³/µL), fasting glucose (mg/dL), serum creatinine (mg/dl), total cholesterol (mg/dL), high-density lipoprotein (HDL) cholesterol (mg/dL), triglycerides (mg/dL). Low-density lipoprotein (LDL) cholesterol (mg/dL) was calculated according to the Friedewald equation. Transthoracic echocardiography was performed by experienced cardiologists following standard recommendations. Echocardiographic parameters included left ventricular ejection fraction (%), interventricular septal thickness (mm), posterior wall thickness (mm), and left ventricular end-diastolic volume indexed to body surface area (mL/m²).

The following parameters were measured at the end of acclimation (T1), during both sessions after 15 (T2) and after 30 min (T3) and at the end of vibration combined with music on Day 2 (T4):

- Systolic and diastolic blood pressure (mmHg) were measured at rest using a validated automated sphygmomanometer, according to current clinical guidelines.
- Peripheral oxygen saturation (SpO₂, %) and heart rate (beats per minute, bpm) were continuously monitored using a pulse oximeter. Respiratory rate (breaths per minute) was assessed at rest by direct observation over a standardized time interval.
- Heart rate variability (HRV) was evaluated using the Garmin HRM-Dual heart rate monitor, which transmits data via both ANT+^®^ and Bluetooth^®^ Low Energy. The device, a soft and adjustable chest strap, captures RR intervals and transmits precise linear heart rate data to an app. This generates activity files with HRV metrics, which are downloadable for further analysis. HRV variables analyzed for the present study included:

- Total Power: Represents overall heart rate variability, calculated as the sum of all frequency components over a given period. Higher values indicate greater variability and, in general, a more flexible and responsive autonomic nervous system.
- High Frequency (0.15–0.40 Hz): Primarily reflects parasympathetic (vagal) activity, associated with relaxation and recovery. Greater high-frequency values suggest a good balance between relaxation and activation, indicating an autonomic nervous system that adapts effectively to varying demands.
- Low Frequency (0.04–0.15 Hz): Reflects both sympathetic and parasympathetic activity. Elevated low-frequency values, especially when greater than high-frequency values, may indicate increased stress or sympathetic nervous system activation.

- Salivary cortisol and cortisone levels were assessed using saliva samples collected at awakening time (T0). Participants were instructed in the standardized saliva collection procedure and asked to collect the sample immediately upon awakening on the session day, using a dedicated collection tube. Participants were instructed to avoid eating, drinking, smoking, or brushing their teeth before sample collection. After collection, samples were transported to the study facility within the same morning and maintained at room temperature until delivery. Upon arrival, saliva samples were immediately stored at − 20 °C until analysis. Cortisol and cortisone concentrations were measured in a single analytical session at the Endocrinology Laboratory of the University of Padua using a validated liquid chromatography–tandem mass spectrometry assay previously reported [26]. The cortisol-to-cortisone ratio was subsequently calculated. A concomitant medication or medical condition that can affect salivary cortisol measurement was an exclusion criterion.
- Perceived stress was measured using a visual stress intensity scale (VSS). The stress intensity scale consisted of a standardized 10 cm white line anchored by extremes (0 = no stress, 10 = maximum stress), on which participants indicated their level of acute stress after acclimatization and after each session.

### Statistical analysis

All collected data were handled confidentially in accordance with Italian data protection law (Decree 196/2003). Each participant was assigned an anonymous ID code. Data were stored in password-protected files and accessed only by study personnel.

Categorical variables were summarized as frequencies and percentages, and continuous variables as means and standard deviations (SD) or medians and interquartile ranges (IQR), as appropriate. Normality of continuous variables was assessed using the Shapiro-Wilk test.

**Table 1:** Clinical characteristics of the study participants (*n* = 11)

Characteristic	Value
Age, years	62.2 ± 6.0 median 60 (56–69); min-max 56–73
Smokers, n (%)	2 (18.2)
BMI, kg/m²	26.8 ± 3.1 median 26.0 (25.1–29.4); min-max 22.1–33.4
Hemoglobin, g/L	154.6 ± 8.4
Platelets, ×10³/µL	257.7 ± 78.4
Glucose, mg/dL	96.1 ± 11.3
Total cholesterol, mg/dL	218.7 ± 39.3
HDL cholesterol, mg/dL	55.2 ± 5.1
Triglycerides, mg/dL	107.1 ± 33.9
LDL cholesterol, mg/dL	132.8 ± 23.9
Creatinine, mg/dL	0.92 ± 0.11
Systolic BP, mmHg	148.2 ± 16.6
Diastolic BP, mmHg	88.2 ± 8.7
Ejection fraction, %	67.7 ± 7.8
Septal thickness, mm	12.0 ± 0.8
Posterior wall thickness, mm	12.1 ± 0.8
End-diastolic volume, ml/m²	43.7 ± 5.7
Use of ACE inhibitors, n (%)	5 (45.5)
Use of sartans, n (%)	4 (36.4)
Use of beta-blockers, n (%)	2 (18.2)
Use of Calcium antagonists, n (%)	2 (18.2)
Use of diuretics, n (%)	2 (18.2)
Use of statins, n (%)	5 (45.5)

BMI, body mass index; BP, blood pressure; SD, standard deviation; Q1, first quartile; Q3, third quartile

**Table 2:** Clinical parameters at different time points

Parameter (unit)	Day 1				Day 2				*p*-value (Day*)	*p*-value (Time*)
	T0	T1	T2	T3	T0	T1	T2	T3		
Systolic BP (mmHg)	–	129.4 ± 10.7	122.3 ± 11.9	123.7 ± 10.5	–	129.6 ± 15.4	123.4 ± 9.0	121.5 ± 8.7	0.3769	0.0167
Diastolic BP (mmHg)	–	81.4 ± 7.1	78.4 ± 8.1	78.9 ± 5.7	–	81.2 ± 8.2	79.2 ± 6.5	77.5 ± 6.8	0.5981	0.1928
SpO₂ (%)	–	94.7 ± 1.7	94.5 ± 1.6	93.9 ± 1.4	–	94.4 ± 1.7	93.6 ± 1.8	93.3 ± 2.1	0.2217	0.0457
HR (bpm)	–	68.4 ± 10.7	69.5 ± 11.1	69.1 ± 11.0	–	71.5 ± 9.1	68.8 ± 8.6	68.9 ± 9.1	0.0857	0.9984
RR (breaths/min)	–	17.0 ± 3.5	14.2 ± 2.1	16.4 ± 4.2	–	16.4 ± 4.5	15.6 ± 2.8	15.3 ± 3.5	0.4058	0.5804
VSS (score)	–	2.5 ± 2.5	–	1.6 ± 0.9	–	2.5 ± 1.9	–	1.1 ± 0.9	0.4478	0.0023

BP, blood pressure; SpO₂, peripheral capillary oxygen saturation; HR, heart rate; RR respiratory rate, VSS visual stress intensity scale. Values are mean ± SD. T0 upon awakening; T1 after 10 min acclimatization; T2 mid-session; T3 end of session. Mixed-effects models with Day and time as fixed effects and repeated measures within subjects were performed

**Table 3:** Hormonal parameters at different time points

Parameter (unit)	Day 1				Day 2				*p*-value (Day*)	*p*-value (Time*)
	T0	T1	T2	T3	T0	T1	T2	T3		
Cortisol (nmol/L)	9.9 ± 4.4	2.8 ± 1.2	2.4 ± 0.7	2.0 ± 0.7	11.0 ± 8.3	3.3 ± 2.2	2.6 ± 1.4	1.9 ± 0.9	0.3702	< 0.0001
Cortisone (nmol/L)	27.9 ± 6.6	17.5 ± 5.4	16.0 ± 3.7	14.7 ± 3.2	27.0 ± 11.8	18.1 ± 5.0	15.8 ± 4.4	13.6 ± 3.5	0.5068	< 0.0001
Cortisol-to-cortisone ratio	0.4 ± 0.1	0.2 ± 0.1	0.2 ± 0.0	0.1 ± 0.0	0.4 ± 0.2	0.2 ± 0.1	0.2 ± 0.1	0.1 ± 0.0	0.7342	< 0.0001

Values are mean ± SD. T0 upon awakening; T1 after 10 min acclimatization; T2 mid-session; T3 end of session. Mixed-effects models with Day and Time as fixed effects and repeated measures within subjects were performed

**Table 4:** Changes of cardiovascular, respiratory, and laboratory parameters during the Day 1 and Day 2 sessions

Parameter (unit)	Day 1	Day 2	Difference (Day 2 vs. Day 1) *p*-value§	Mixed models	
				Day *p*-value*	Time *p*-value*
Systolic BP (mmHg)	-5.64 ± 6.96	-8.09 ± 10.47	0.4223	0.6854	0.0062
Diastolic BP (mmHg)	-2.45 ± 3.05	-3.64 ± 5.07	0.5200	0.5599	0.0269
SpO₂ (%)	-0.82 ± 1.78	-1.09 ± 1.64	0.6827	0.2626	0.0371
HR (bpm)	0.73 ± 6.98	-2.64 ± 4.23	0.1832	0.4221	0.6083
RR (breaths/min)	-0.64 ± 4.15	-1.09 ± 3.62	0.8124	0.3116	0.3116
VSS (score)	-0.91 ± 1.92	-1.36 ± 1.69	0.1762	0.3952	0.0043
Cortisol (nmol/L)^	-0.82 ± 0.83	-1.39 ± 1.53	0.3050	0.4586	0.0042
Cortisone (nmol/L)^	-2.80 ± 4.44	-4.54 ± 2.18	0.6926	0.9788	0.0037
Cortisol-to-cortisone ratio^	-0.02 ± 0.05	-0.03 ± 0.05	0.3966	0.6051	0.0344

Values represent changes (T3–T1) and are reported as mean ± SD. T1 after 10 min of acclimatization; T3 end of control or treatment session. ^Values rapresent changes (T2-T0) and are reported as mean ± SD. § Paired t-test or Wilcoxon signed-rank test comparing Day 2 versus Day 1 changes. * p-values derived from mixed-effects models for a single-sequence cross-over design, with Day and time as fixed effects and repeated measures within subjects. SD standard deviation, BP blood pressure, SpO₂ oxygen saturation, HR heart rate, RR respiratory rate, VSS visual stress intensity scale

**Table 5:** Changes in heart rate variability parameters during the Day 1 and Day 2 sessions

HRV parameter (unit)	Day 1 T1–T3 (mean ± SD)	Day 2 T1–T3 (mean ± SD)	Day 2 T4 (mean ± SD)	Day 1 vs. Day 2 (T1–T3) *p*-value§	Day 2 change (T1–T3) vs. T4 *p*-value§	Mixed models*	
						Day *p*-value	Time *p*-value
Total power (ms^2^)	2996.4 ± 2007.9	2433.8 ± 1115.1	1641.8 ± 1982.1	0.5150	0.4933	0.3800	0.6847
Low-frequency power (ms^2^)	1855.6 ± 1380.6	1693.2 ± 734.7	1177.6 ± 1574.1	0.8147	0.5607	0.6131	0.6948
High-frequency power (ms^2^)	1140.8 ± 754.1	740.6 ± 434.3	464.3 ± 426.2	0.2243	0.3894	0.1049	0.7305

Values represent changes from T1 to T3 and are reported as mean ± SD. T1 after 10 min of acclimatization; T3 end of control or treatment session; T4 after 7 min of music plus vibration. § Paired t-test or Wilcoxon signed-rank test on within-subject differences, as appropriate. * p-values derived from mixed-effects models for a single-sequence cross-over design, with Day and time as fixed effects and repeated measures within subjects. Models were adjusted for LDL, interventricular septal thickness, ACE inhibitor/ARB use, calcium antagonist use, and Day 1 baseline values. SD standard deviation, HRV heart rate variability

For clinical, cardiorespiratory, hormonal (cortisol, cortisone, cortisol-to-cortisone ratio), and stress-related outcomes with repeated measures over time, changes over time, and differences between sessions were analyzed using mixed-effects models. In these models, Day (Day 1 vs. Day 2) and time (T1, T2, T3, T4, and T0 for cortisol) were included as fixed effects, with subject treated as a repeated factor to account for within-subject correlation. These analyses provided p-values for the main effects of Day and time. The p-value for the effect of time reflects changes across the different measurement points within each session, whereas the p-value for the effect of Day reflects overall differences between the control (Day 1) and intervention (Day 2) sessions, independently of time.

Within-session changes were calculated as the difference between end-of-session and post-acclimatization values (T3-T1) for physiological and stress-related parameters, and as differences between post-session and awakening values for hormonal parameters. These changes were compared between Day 1 and Day 2 using paired t-tests or Wilcoxon signed-rank tests, depending on the data distribution, as indicated in the tables. Heart rate variability variables were analyzed separately for the control session and the vibration-only or vibration-plus-music phases. Changes from baseline and differences between intervention phases were evaluated using paired comparisons and mixed-effects models, as appropriate. When specified, mixed-effects models were also adjusted for relevant covariates, including LDL cholesterol, interventricular septal thickness, use of antihypertensive medications (ACE/ARBs and calcium channel blockers), and the baseline value of the outcome variables.

All tests were two-sided, and a p-value < 0.05 was considered statistically significant. Given the exploratory nature of this study, no adjustment for multiple comparisons was considered. Statistical analysis was performed using SAS software, version 9.4 (SAS Institute Inc., Cary, NC, USA).

## Results

### Participant characteristics

A total of 11 male participants (median age 60 years, IQR 56–69; range 56–73) with mild to moderate hypertension were included. The main characteristics of the sample are shown in Table 1. Two participants (18.2%) were active smokers, and the mean BMI was 26.8 (SD 3.1) kg/m². Mean fasting glucose was 96.1 (SD 11.3) mg/dL, and mean LDL cholesterol was 132.8 (SD 23.9) mg/dL. Baseline systolic and diastolic BP were 148.2 (SD 16.6) mmHg and 88.2 (SD 8.7) mmHg, respectively. Echocardiography showed a normal left ventricular ejection fraction, averaged 67.7% (SD 7.8), and mildly increased left ventricular wall thickness, indicating an early hypertensive cardiac remodeling.

Regarding pharmacological treatment, nearly half of the participants were receiving ACE inhibitors (45.5%) or statins (45.5%). A smaller proportion was treated with sartans (36.4%), calcium channel blockers (18.2%), or diuretics (18.2%), while 2 participants were receiving beta-blockers (18.2%) at the time of enrollment.

### Cardiorespiratory changes

Measurements at different time points are described in Tables 2 and 3, while Table 4 reports the changes in cardiovascular, respiratory, and laboratory parameters during the Day 1 and Day 2 sessions. Between T1 and T3, systolic BP decreased by − 5.64 (SD 6.96) mmHg on Day 1 and by − 8.09 (SD 10.47) mmHg on Day 2. The reduction from T1 and T3 was not significant (*p* = 0.17), but no substantial differences were observed between Day 1 and 2 (*p* = 0.377). Diastolic BP declined by − 2.45 (SD 3.05) mmHg (Day 1) and − 3.64 (SD 5.07) mmHg, with no significant differences by assessment time or Day.

Heart rate on average increased by 0.73 (SD 6.98) bpm on Day 1 and decreased by 2.64 (SD 4.23) bpm on Day 2, with a marginal non-significant interaction with the Day of assessment (*p* = 0.086). Similar slight decreases in respiratory rate were observed during the sessions on Day 1 and Day 2, as well as in SpO₂ values.

### Stress-related parameters’ changes

Both on Day 1 and Day 2, VSS significantly decreased during the session − 0.91 (SD 1.92) points on Day 1; − 1.36 (SD 1.69) points on Day 2; (*p* = 0.002). Cortisol levels decreased by − 0.82 (SD 0.83) nmol/L (Day 1) and − 1.39 (SD 1.53) nmol/L (Day 2); (*p* = 0.3050). Significant reductions from T1 to T3 were also observed in salivary cortisone levels − 2.80 (SD 4.44) nmol/L on Day 1; − 4.54 (SD 2.18) nmol/L on Day 2; (*p* < 0.001), and the cortisol/cortisone ratio (Table 2). However, these parameters did not differ significantly between the two sessions.

### Heart rate variability

Analysis of HRV data from the Garmin HRM-Dual (Table 5) showed that total power was higher on Day 1 (2996.4 [SD 2007.9] ms²) compared to vibration only (2433.8 [SD 1115.1] ms²), and the combined vibration and music phase 1641.8 (SD 1982.1) ms² on Day 2. Low-frequency power followed a similar pattern, with values of 1855.6 (SD 1380.6) ms² on Day 1, 1693.2 (SD 734.7) ms² during vibration only, and 1177.6 (SD 1574.1) ms² during vibration plus music. High-frequency power, reflecting parasympathetic activity, was also higher on Day 1 (1140 [SD 754.1] ms²), decreasing to 740.6 (SD 434.3) ms² with vibration only and 464.3 (SD 426.2) ms² during the combined phase. None of these differences reached statistical significance, neither considering changes over time nor between Day 1 and Day 2.

## Discussion

This pilot cross-over study investigated the acute effects of VAS on cardiovascular and stress-related parameters in male adults with mild to moderate hypertension. Although no statistically significant differences were observed between the intervention and control conditions, several consistent trends emerged, suggesting potential physiological relevance.

In the present study, we selected a low-frequency protocol centered on 40 Hz, as this frequency has been repeatedly used in vibroacoustic applications and was confirmed to be feasible and well tolerated while providing a clearly perceivable, rhythmically stable stimulus, in line with previous research [27]. The session duration was chosen to match commonly adopted clinical protocols (approximately 10–45 min across studies) and to allow detection of acute physiological responses without imposing excessive burden on participants.

When comparing the pre- and post-session assessments, we found that systolic and diastolic BP, heart rate, respiratory rate, and perceived stress all showed greater reductions during the VAS session compared to the control session. While these differences did not reach statistical significance, likely due to the small sample size, the direction and consistency of change across multiple parameters align with previous findings on the potential autonomic and stress-reducing effects of both music therapy and low-frequency vibration. Importantly, the convergence of trends across independent domains (hemodynamic, respiratory, endocrine, and subjective stress measures) strengthens the biological plausibility of an acute relaxation-related response.

HRV is widely recognized as a non-invasive marker of autonomic nervous system flexibility and cardiovascular health. Higher resting HRV is generally associated with enhanced parasympathetic (vagal) control and a favorable cardiovascular prognosis. For this reason, HRV is often interpreted as an index of physiological resilience and adaptive capacity. However, experimental studies investigating the psychophysiological effects of music have reported seemingly paradoxical findings. In particular, several studies in the field of music cognition and affective neuroscience have documented acute reductions in HRV during music listening, especially when the music is emotionally salient or aesthetically engaging. Notably, research by Koelsch and colleagues [28] has shown that music-induced emotional experiences may be accompanied by decreased HRV, despite the generally positive emotional valence of the stimulus. In our study, HRV was higher in the absence of stimulation than with vibration alone, and the lowest values were observed during the combined vibration and music phase. This apparent contradiction raises important conceptual questions regarding the interpretation of HRV changes in response to short-term emotional and cognitive stimulation. We need to clarify how transient reductions in HRV during music listening can be reconciled with HRV’s established role as a marker of cardiovascular and autonomic health. One possible interpretation is that short-term HRV reductions may reflect rhythmic entrainment and increased attentional/emotional engagement rather than an adverse autonomic shift; thus, acute HRV responses to stimulation should be interpreted in the context of this experimental condition and time scale.

The observed reductions in salivary cortisol and cortisone levels during the VAS session are consistent with a stress-relieving effect. Overall, if confirmed by larger studies, these findings suggest that VAS may acutely influence cardiovascular and neuroendocrine stress markers [29]. Limitations of the study include the small sample size and the inclusion of only adult males, which, therefore, limit generalizability to women and broader hypertensive populations. Moreover, the study had a single-session cross-over design, in which each participant served as his own control. On the one hand, this design may reduce between-subject variability and is well-suited for detecting short-term physiological effects in small samples. On the other hand, the actual effectiveness of VAS intervention compared with usual care or selected controls could be more appropriately estimated through randomized controlled trials. Moreover, no sham vibration control was used, which may have introduced expectancy bias. In addition, participants and investigators were not blinded to condition, and day-to-day variability (e.g., sleep quality, caffeine intake, anticipatory stress) may have contributed noise to short-term physiological measures. Because music preference and familiarity can influence autonomic and endocrine responses, the use of a fixed protocol may have introduced inter-individual variability that could not be fully controlled. Finally, salivary cortisol and cortisone exhibit circadian variation; although time points were standardized within sessions, residual diurnal effects cannot be entirely excluded.

Instead, the use of an innovative tool to administer VAS, the large set of cardiorespiratory parameters, and the collection of stress-related data represent strengths of the study. Additional strengths include the multimodal assessment combining objective physiological measures (BP, cardiorespiratory indices, HRV) with neuroendocrine markers and subjective stress ratings, providing a more comprehensive picture of acute stress regulation.

Future studies should use larger cohorts and longer intervention periods to determine whether acute effects can be sustained over time. Incorporating sham vibration and/or music-only arms, along with blinding where feasible, would help disentangle specific versus nonspecific effects and reduce expectancy bias. Finally, repeated-session interventions with clinically meaningful endpoints (ambulatory BP, longer-term HRV profiles, and patient-reported outcomes) will be essential to evaluate potential therapeutic relevance.

## Conclusion

VAS was feasible and well tolerated in men with mild-to-moderate hypertension, with no relevant adverse events. While between-session differences did not reach statistical significance in this pilot study, the directionally consistent reductions in hemodynamic and neuroendocrine stress markers suggest that combined vibrotactile and auditory stimulation may acutely modulate autonomic regulation and stress-system activity. These findings support the rationale for adequately powered controlled trials to confirm efficacy, define optimal stimulation parameters (frequency, duration, and dose), and determine whether repeated VAS exposure can translate into clinically meaningful improvements in blood pressure control and stress burden.

## Data Availability

No datasets were generated or analysed during the current study.
